# Salt-Sensitive Hypertension Induces Osteoclastogenesis and Bone Resorption *via* Upregulation of Angiotensin II Type 1 Receptor Expression in Osteoblasts

**DOI:** 10.3389/fcell.2022.816764

**Published:** 2022-04-04

**Authors:** Adya Pramusita, Hideki Kitaura, Fumitoshi Ohori, Takahiro Noguchi, Aseel Marahleh, Yasuhiko Nara, Ria Kinjo, Jinghan Ma, Kayoko Kanou, Yukinori Tanaka, Itaru Mizoguchi

**Affiliations:** ^1^ Division of Orthodontics and Dentofacial Orthopedics, Tohoku University Graduate School of Dentistry, Sendai, Japan; ^2^ Division of Dento-Oral Anesthesiology, Tohoku University Graduate School of Dentistry, Sendai, Japan

**Keywords:** angiotensin II type 1 receptor, mouse, osteoblast, osteoclast, salt-sensitive hypertension, TNF-α

## Abstract

Hypertension is a chronic-low grade inflammatory disease, which is known to be associated with increased bone loss. Excessive activity of the local renin–angiotensin system (RAS) in bone leads to increased bone resorption. As inflammatory cytokines may activate RAS components, we hypothesized that the elevated proinflammatory cytokine levels in hypertension activate bone RAS and thus lead to increased bone resorption. To investigate whether salt-sensitive hypertension (SSHTN) induces osteoclastogenesis and bone resorption, we generated a model of SSHTN in C57BL/6J mice by post-*N* ^ω^-nitro-l-arginine methyl ester hydrochloride (l-NAME) high-salt challenge. SSHTN led to the reduction of distal femur trabecular number and bone volume fraction, while trabecular separation of femoral bone showed a significant increase, with no change in cortical thickness. Histomorphometric examination showed a significant reduction in trabecular bone volume fraction with an increased number of multinucleated tartrate-resistant acid phosphatase (TRAP)-positive cells and increased osteoclast surface fraction in the trabecular distal femur of hypertensive mice. Furthermore, analysis of gene expression in bone tissue revealed that TRAP and RANKL/OPG mRNA were highly expressed in hypertensive mice. TNF-α and angiotensin II type 1 receptor (AGTR1) mRNA and protein expression were also upregulated in SSHTN mice. These observations suggested that TNF-α may have an effect on AGTR1 expression leading to osteoclast activation. However, TNF-α stimulation did not promote AGTR1 mRNA expression in osteoclast precursors in culture, while TNF-α increased AGTR1 mRNA expression in osteoblast culture by activation of downstream p38. Angiotensin II was also shown to increase TNF-α-induced RANKL/OPG mRNA expression in primary osteoblast culture and osteoclastogenesis in a TNF-α-primed osteoblast and osteoclast precursor co-culture system. In addition, local injection of lipopolysaccharide into the supracalvariae of SSHTN mice markedly promoted osteoclast and bone resorption. In conclusion, mice with SSHTN show increased osteoclastogenesis and bone resorption due mainly to increased TNF-α and partly to the upregulation of AGTR1 in osteoblasts.

## 1 Introduction

Hypertension is a major global health problem due to its high prevalence and because it is one of the most important risk factors for cardiovascular, cerebrovascular, and chronic kidney diseases, which are the leading causes of death worldwide (Ettehad et al., 2016; Li et al., 2016; Xie et al., 2016; Arvanitakis et al., 2018). The number of adults with hypertension is increasing, with worldwide estimated prevalence rates of 26.4% (corresponding to 972 million adults) in 2000 and 31.1% (corresponding to 1.39 billion adults) in 2010 (Kearney et al., 2005; Mills et al., 2016). Moreover, the global burden of hypertension is expected to increase by 60% reaching approximately 1.56 billion adults by 2025 (Kearney et al., 2005).

The systemic renin–angiotensin system (RAS) has been widely investigated because of its roles in the regulation of blood pressure, electrolyte homeostasis, and the development of hypertension (Ahn et al., 2017; Han et al., 2017). Several human studies suggested that hypertension is associated with low bone mineral density and increased risk of fracture (Li et al., 2017; Lian et al., 2017; Ye et al., 2017). In addition to systemic RAS, many tissues, including bone, have a local RAS the excessive activity of which leads to increased bone resorption and decreased bone formation (Shimizu et al., 2008; Asaba et al., 2009; Zhou et al., 2017). In a previous study, RAS components were expressed in the femora of both sham and ovariectomized (OVX) rats, and the levels of angiotensin II, angiotensin II type 1 receptor (AGTR1), and angiotensin converting enzyme (ACE) protein expression were shown to be upregulated in the OVX group, while OVX-induced bone loss was ameliorated by 6 weeks of oral losartan treatment, an inhibitor of AGTR1 (Abuohashish et al., 2017c).

Inflammation is known to be closely related to hypertension. Recent animal studies have shown that hypertension is associated with elevated plasma levels of proinflammatory cytokines, such as C-reactive protein (CRP), interleukin (IL)-6, IL-1β, and tumor necrosis factor (TNF)-α (Lu et al., 2016; Liu et al., 2019; Dai et al., 2020). Consistent with the results of animal studies, a number of clinical studies have also shown that patients with hypertension commonly have elevated plasma concentrations of proinflammatory cytokines compared to normotensive patients (Jiménez et al., 2016; Pouvreau et al., 2018; Chen et al., 2019; Jayedi et al., 2019). In addition, there is emerging evidence that proinflammatory cytokines activate RAS components. Both IL-1β and TNF-α were shown to induce AGTR1 expression in cultured cardiac fibroblasts and chondrocytes (Peng et al., 2002; Gurantz et al., 2005; Tsukamoto et al., 2014). Moreover, the osteoclastogenic cytokine, TNF-α, is a potent stimulator of osteoclast activity, and plays pivotal roles in bone metabolism and under pathological conditions in bone diseases. TNF-α was shown to directly promote the differentiation of osteoclast precursors into multinucleated tartrate-resistant acid phosphatase (TRAP)-positive osteoclasts in the presence of monocyte colony-stimulating factor (M-CSF) and in the absence of receptor activator of nuclear factor-κB ligand (RANKL) (Kishikawa et al., 2019; Noguchi et al., 2020; Ohori et al., 2020). On the other hand, TNF-α has also been shown to stimulate the production of M-CSF and RANKL in osteoblast-like cells (Xiong et al., 2014).

Lipopolysaccharide (LPS), a major cell-surface antigen of gram-negative bacteria, acts as a potent stimulator of inflammation and osteolytic bone loss (Abu-Amer et al., 1997; Sakuma et al., 2000). LPS has been shown to induce the release of several proinflammatory cytokines and factors, including TNF-α, IL-1, prostaglandin E2 (PGE2), and RANKL, from fibroblasts, macrophages, osteoblasts, and other cell types at inflammatory sites. Daily subcutaneous supracalvarial injection of LPS was also reported to induce osteoclastogenesis and bone resorption due to increases in levels of RANKL and TNF-α (Shen et al., 2018; Shima et al., 2018; Kishikawa et al., 2019). However, there have been no previous studies regarding the effects of salt-sensitive hypertension (SSHTN) on inflammation-induced osteoclastogenesis and bone resorption.

Based on the observations outlined above, we hypothesized that elevated level of the proinflammatory cytokine, TNF-α, under conditions of hypertension may promote bone RAS activation, resulting in the induction of osteoclastogenesis and bone resorption. To test this hypothesis, we generated a mouse model of SSHTN by post-*N* ^ω^-nitro-l-arginine methyl ester hydrochloride (l-NAME) high-salt challenge. Here, we found that SSHTN mice exhibited marked increases in osteoclast number and bone resorption area due mainly to increased TNF-α and partly to the upregulation of AGTR1 in osteoblasts.

## 2 Materials and Methods

### 2.1 SSHTN Mouse Model and Reagents

**FIGURE 1:**
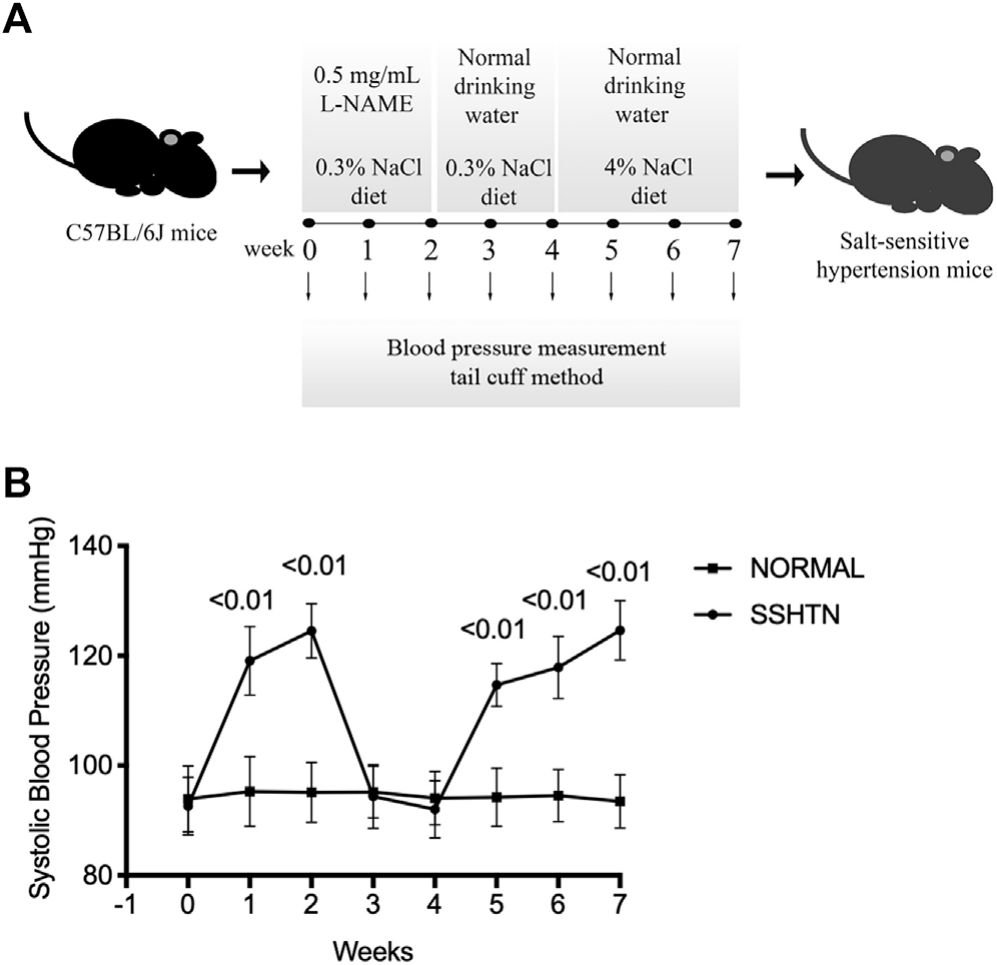
Systolic blood pressure. **(A)** The experimental protocol to generate SSHTN mouse model consisted of three consecutive periods: a 2-weeks nitric oxide synthesis inhibition period with l-NAME, followed by a 2-weeks washout period, and subsequent high-salt diet for 3 weeks. **(B)** SBP was measured in normal control mice and SSHTN mice (*n* = 4).

Male C57BL/6J mice, 7–10 weeks old, were purchased from CLEA Japan (Tokyo, Japan). To generate the SSHTN model, mice were administered l-NAME (0.5 mg/mL; Sigma-Aldrich, St. Louis, MO, United States) in their drinking water for 2 weeks to inhibit nitric oxide synthesis, followed by a 2-weeks washout period, and a 3-weeks exposure to a high-salt diet (CE-2 containing 4% NaCl; CLEA Japan) (Lopez Gelston et al., 2018) (Figure 1A). Normal control mice received tap water and standard diet for 7 weeks. All diets and water were provided ad libitum. Systolic blood pressure (SBP) was monitored weekly by tail-cuff plethysmography using a blood pressure monitor (MK-1030; Muromachi Kikai Co., Tokyo, Japan) in previously trained mice. All animal care and experiments were approved by Tohoku University of Science Animal Care and Use Committee. LPS from *Escherichia coli*, angiotensin II, and olmesartan (angiotensin II type 1 receptor blocker) were purchased from Sigma-Aldrich. Recombinant murine TNF-α was prepared in our laboratory as described previously (Kitaura et al., 2004).

### 2.2 Histological Analysis

The femora of normal control and SSHTN mice were resected immediately after sacrifice, fixed in 4% formaldehyde in phosphate-buffered saline (PBS), and then demineralized in 14% ethylenediaminetetraacetic acid (EDTA) for 5 weeks. After dehydration through a graded ethanol series, they were embedded in paraffin and cut into sections 5 μm thick. Hematoxylin and eosin (H&E) and tartrate-resistant acid phosphatase (TRAP) staining were performed as described previously (Nara et al., 2020) to evaluate trabecular bone volume fraction (BV/TV, %), osteoclast surface fraction (Oc.S/BS, %), and the number of TRAP-positive cells (i.e., osteoclasts) per bone perimeter (N.Oc/B.Pm, 1/mm) using ImageJ software (NIH, Bethesda, MD, United States). TRAP-positive cells containing three or more nuclei were considered to be osteoclasts. The regions of interest (ROIs; 250 μm × 250 μm) were limited to trabecular bone and extended 250 μm proximal to the distal growth plate to exclude primary spongiosa, new bone growth, and cortical bone.

In addition, osteoclastogenesis was induced by subcutaneous supracalvarial injection of LPS at a dose of 100 μg/day for 5 days as described previously (Ishida et al., 2019). All animals were randomly divided into five groups as follows: normal controls with daily subcutaneous injection of PBS, 10 μg/day LPS, or 100 μg/day LPS; SSHTN mice with daily subcutaneous injection of PBS or 10 μg/day LPS. Calvariae were resected on day 6, decalcified in 14% EDTA for 3 days, embedded in paraffin, and cut into sections 5 μm thick perpendicular to the sagittal suture. TRAP and hematoxylin counterstaining were performed as described previously (Ishida et al., 2019). The number of osteoclasts located in the mesenchyme of the sagittal suture was counted in all slides as described previously (Ishida et al., 2019). All sections were observed and photographed using a light microscope (Olympus DP72; Olympus Co., Ltd., Tokyo, Japan).

### 2.3 Cell Culture

Osteoclast precursors were obtained as described previously (Marahleh et al., 2019). Briefly, bone marrow cells were flushed from the femora and tibiae of male C57BL/6J mice into sterile 6-cm culture dishes with α-modified minimal essential medium (α-MEM; Wako, Osaka, Japan) using a 30-gauge needle. The bone marrow was then filtered through a 40-μm nylon cell strainer (Falcon, Corning, NY, United States) and centrifuged. The harvested cells were incubated in α-MEM containing 10% fetal bovine serum (FBS) (Biowest, Nuaillé, France), 100 IU/ml penicillin G, 100 μg/ml streptomycin (Wako), and M-CSF for 3 days. Non-adherent cells were removed by washing with PBS and adherent cells were harvested using trypsin-EDTA solution (Gibco; Thermo Fisher Scientific, Inc., Waltham, MA, USA). These cells were used as osteoclast precursors in this study.

Primary osteoblasts were isolated from the calvariae of neonatal C57BL/6J mice as described previously (Shima et al., 2018). Briefly, calvariae were incubated with 0.2% (w/v) collagenase in isolation buffer (70 mM NaCl, 10 mM NaHCO_3_, 60 mM sorbitol, 3 mM K_2_HPO_4_, 1 mM CaCl_2_, 0.1% (w/v) BSA, 0.5% (w/v) glucose, and 25 mM HEPES) for 20 min at 37°C with agitation at 300 rpm (fraction 1), followed by digestion with 5 mM EDTA (Wako) in PBS containing 0.1% BSA (Sigma-Aldrich) for 15 min at 37°C with agitation at 300 rpm (fraction 2), then incubated in collagenase for 20 min twice to obtain fraction 3 and fraction 4. Cells from fraction 2–4 were collected as the osteoblast-rich fraction (Ohori et al., 2019; Asano et al., 2021).

To investigate the effects of angiotensin II on priming of osteoblasts by TNF-α, osteoblasts (1 × 10^4^ cells) were cultured in the presence or absence of TNF-α for 24 h, and then washed three times with PBS to remove TNF-α. Osteoclast precursors (5 × 10^4^ cells) were then added on top of the osteoblasts in 96-well plates and cultured in α-MEM with 10^–8^ M 1.25(OH)_2_D_3_ + 10^–6^ M PGE_2_ (both from Sigma-Aldrich), 10^–8^ M 1.25(OH)_2_D_3_ + 10^–6^ M PGE_2_ + 10^–6^ M angiotensin II, 10^–8^ M 1.25(OH)_2_D_3_ + 10^–6^ M PGE_2_ + 10^–6^ M angiotensin II + 10^–5^ M angiotensin II type 1 receptor blocker or without additional agents as controls for 3 days. Cells were fixed with 4% formaldehyde and incubated with TRAP solution consisting of acetate buffer (pH 5.0), naphthol AS-MX phosphate, fast red violet LB salt, and 50 mM sodium tartrate at 37°C for 30 min. TRAP-positive multinucleated osteoclasts ≥150 µm in diameter with three or more nuclei were visualized and counted manually under a light microscope as described previously (Nara et al., 2020).

### 2.4 Real-Time RT-PCR Analysis

For real-time RT-PCR analysis, tibiae were crushed using a cell disrupter (Micro Smash MS-100R; Tomy Seiko, Tokyo, Japan) in 800 μL of TRIzol reagent (Invitrogen, Carlsbad, CA, United States). For *in vitro* experiments, osteoclast precursors and osteoblasts were cultured in serum-free medium overnight, followed by incubation in culture medium supplemented with TNF-α (100 ng/mL) or PBS as controls for 24 h. Moreover, to examine whether an increase in AGTR1 mRNA expression level in TNF-α-primed osteoblast affected the levels of RANKL and OPG mRNA expression, primary osteoblasts were cultured in culture medium supplemented with PBS or TNF-α (100 ng/mL) for 24 h followed by changing the medium to remove TNF-α. Osteoblasts were then exposed to 10^–6^ M angiotensin II for 24 h. Total RNA was extracted from cells and tissue samples using a RNeasy Mini Kit (Qiagen, Hilden, Germany). cDNA was synthesized using Superscript IV reverse transcriptase (Invitrogen) with the same amount of total RNA. The levels of TNF-α, IL-1β, AGTR1, ACE, TRAP, RANKL, OPG, and glyceraldehyde 3-phosphate dehydrogenase (GAPDH) gene expression were measured using TB Green Premix Ex Taq II (Takara, Shiga, Japan) and a thermal cycler (Dice Real Time System; Takara) under the following conditions: initial denaturation at 95°C for 30 s followed by 50 cycles of denaturation at 95°C for 5 s and annealing at 60°C for 30 s, and final dissociation stage. Relative expression levels of target mRNAs were calculated by normalization relative to GAPDH mRNA. The primer sequences were as follows: 5′-GGT​GGA​GCC​AAA​AGG​GTC​A-3′ and 5′-GGG​GGC​TAA​GCA​GTT​GGT-3′ for GAPDH; 5′-CTG​TAG​CCC​ACG​TCG​TAG​C-3′ and 5′-TTG​AGA​TCC​ATG​CCG​TTG-3′ for TNF-α; 5′-CTC​AAC​TGT​GAA​ATG​CCA​CC-3′ and 5′-TGT​CCT​CAT​CCT​GGA​AGG​T-3′ for IL-1β; 5′-AGT​CGC​ACT​CAA​GCC​TGT​CT-3′ and 5′-ACT​GGT​CCT​TTG​GTC​GTG​AG-3′ for AGTR1; 5′-CCA​CTA​TGG​GTC​CGA​GTA​CAT​CAA-3′ and 5′-AGG​GCG​CCA​CCA​AAT​CAT​AG-3′ for ACE; 5′-AAC​TTG​CGA​CCA​TTG​TTA-3′ and 5′-GGG​GAC​CTT​TCG​TTG​ATG​T-3′ for TRAP; 5′-CCT​GAG​GCC​CAG​CCA​TTT-3′ and 5′-CTTGGCCCAGCCTCGAT-3′ for RANKL; and 5′ -ATC​AGA​GCC​TCA​TCA​CCT​T-3′ and 5′ -CTT​AGG​TCC​AAC​TAC​AGA​GGA​AC- 3′ for OPG.

### 2.5 Western Blotting Analysis

To investigate the effects of TNF-α on ERK1/2, p38, JNK mitogen-activated protein kinases (MAPKs), and IκBα phosphorylation in osteoblasts, primary osteoblasts were cultured in 60-mm cell culture dishes (Corning) in serum-free medium for 3 h. TNF-α (100 ng/mL) was then added to the dishes for specific periods (0, 5, 15, 30, 60 min). To examine the effects of MAPKs and nuclear factor (NF)-κB in the regulation of AGTR1 protein expression, TNF-α-pretreated osteoblasts were preincubated with 10 μM p38 MAPK inhibitor (InSolution™ SB 203580; EMD Millipore, Billerica, MA, United States), 10 μM ERK1/2 inhibitor (InSolution™ U0126; EMD Millipore), 10 μM JNK inhibitor (InSolution™ JNK Inhibitor II; EMD Millipore), and 10 μM NF-κB inhibitor (BAY11-7082; Sigma-Aldrich), and then exposed to TNF-α (100 ng/mL) for 24 h. The distal femur and cells were extracted using radioimmunoprecipitation assay (RIPA) lysis buffer (Millipore, Burlington, MA, United States) containing 1% protease and phosphatase inhibitor (Thermo Fisher Scientific). Total protein concentrations were quantified using a Pierce BCA protein assay kit (Thermo Fisher Scientific). Protein was treated with β-mercaptoethanol (Bio-Rad, Hercules, CA, United States) and Laemmli sample buffer (Bio-Rad) and denatured at 95°C for 5 min prior to SDS-PAGE. Equal amounts of protein were loaded onto 4–15% Mini-PROTEAN TGX Precast Gels (Bio-Rad) and transferred to a PVDF Trans-Blot Turbo Transfer System (Bio-Rad). The membranes were blocked in Block-Ace (DS Pharma Biomedical, Osaka, Japan) for 1 h at room temperature and incubated with antibodies against phospho-p38 MAPK (Thr180/Tyr182) (D3F9) XP, phospho-SAPK/JNK (Thr183/Tyr185) (98F2), phospho-p44/42MAPK (ERK1/2) (Thr202/Tyr204), phospho-IκBα (Ser32) (14D4) (monoclonal rabbit IgG, 1:1,000; Cell Signaling Technology, Danvers, MA, United States), AGTR1 (polyclonal rabbit IgG, 1:1,000; Proteintech Group, Inc., Chicago, IL, United States), TNF-α (polyclonal rabbit IgG, 1:500; GTX110520, Funakoshi, Japan), and β-actin (monoclonal mouse IgG, 1:1,000; Sigma-Aldrich) overnight at 4°C. The membranes were washed in Tris-buffered saline with Triton X-100 (TBS-T) and Tris-buffered saline (TBS), and then incubated with HRP-conjugated anti-rabbit IgG antibody (1:1,000–1:5,000; Cell Signaling Technology) or anti-mouse antibody (1:3,000–1:10,000; GE Healthcare, Chicago, IL, United States) for 1 h at room temperature. Bound antibodies were detected with SuperSignalWest Femto Maximum Sensitivity Substrate (Thermo Fisher Scientific) and a FUSION-FX7. EDGE Chemiluminescence Imaging System (Vilber Lourmat, Collégien, France).

### 2.6 Micro-Computed Tomography

Micro-computed tomography (CT) was performed using ScanXmate-E090 (Comscan, Kanagawa, Japan). Formalin-fixed femora were scanned with isotropic voxel size of 20 μm, x-ray tube voltage of 90 kV, x-ray tube current of 91 μA, with acquisition of 600 projections over 360° rotation, and reconstructed with 1,024 × 1008-pixels matrices. The calvariae were scanned under the same conditions as femora except using a voxel size of 60 μm, x-ray tube voltage of 60 kV, and x-ray tube current of 85 μA, and were reconstructed with 512 × 504-pixels matrices. After image acquisition, three-dimensional images were reconstructed to measure the morphometric parameters using TRI/3D-BONE64 software (RATOC System Engineering, Tokyo, Japan). To analyze the trabecular bone microarchitecture, the ROI for the distal femur began 1 mm proximal to the growth plate and extended 0.5 mm proximally. For cortical bone analysis, 0.5 × 4 mm region was selected in the midshaft centered at 50% of the total femoral length. The standard trabecular morphometric parameters determined were trabecular BV/TV (%), trabecular thickness (Tb.Th, μm), trabecular number (Tb.N, 1/mm), trabecular separation (Tb.Sp, μm), and bone surface area to volume ratio (BS/BV, 1/mm). The cortical morphometric parameter calculated was cortical thickness (Ct.Th, μm). Moreover, a rectangular region of 50 × 70 pixels cantered at the frontal suture was selected to quantify area of destruction in the calvariae using ImageJ (NIH) (Ishida et al., 2019).

### 2.7 ELISA Assay for TNF-α

Serum samples were obtained from normal control and SSHTN mice. TNF-α concentration was measured using ELISA MAX Standard Set Mouse TNF-α (Biolegend, United States) according to the manufacturer’s protocols. 96-well ELISA plates (Biolegend) were precoated with monoclonal hamster antibody in carbonate buffer and incubated overnight at 4°C. To block non-specific binding and reduce background, the plates were incubated for 1 h at room temperature with the addition of 1% bovine serum albumin (BSA; Sigma-Aldrich) in PBS. Diluted mouse TNF-α standard and serum samples were added to the wells and incubated for 2 h at room temperature. Biotinylated goat polyclonal anti-mouse TNF-α detection antibody was added to the wells with 1 h incubation at room temperature. The bound IgG was detected by incubation with HRP-conjugated avidin, followed by colorimetric detection with TMB substrate solution (Biolegend). The reaction was stopped with 2 N H_2_SO_4_ after 30 min, and the absorbance was measured at 450 nm using a microplate reader (Remote Sunrise, Tecan, Japan). Absorbance value at 595 nm was used as a reference.

### 2.8 Statistical Analysis

All results are shown in box plots with the median, interquartile range (IQR), and individual data points. Data were analyzed using GraphPad Prism 9.0 (GraphPad Software, Inc. La Jolla, CA, United States). The distribution of variables was evaluated using the Shapiro–Wilk test. Student’s *t* test or the Mann–Whitney test was used to compare means between two groups, while differences between all groups were examined by one-way analysis of variance (ANOVA) and Tukey’s multiple comparison test or Kruskal–Wallis and Dunn’s multiple comparison test. In all analyses, *p* < 0.05 was taken to indicate statistical significance.

## 3 Results

### 3.1 Post l-NAME High-Salt Challenge Resulted in Increased Systolic Blood Pressure

To induce salt sensitivity, mice were administered l-NAME in their drinking water for 2 weeks, followed by a 2-weeks washout period, and then 3 weeks of exposure to high-salt diet (Figure 1A). Consistent with a previous study (Lopez Gelston et al., 2018), after 2 weeks of l-NAME administration, SBP in l-NAME-treated mice increased to 125 ± 5 mmHg compared to non-treated mice (95 ± 6 mmHg, *p* < 0.01). The SBP of l-NAME-treated mice returned to normal after the 2-weeks washout period (92 ± 5 mmHg). After 3 weeks of salt loading, l-NAME-treated mice showed salt-induced hypertension (125 ± 5 mmHg vs. 93 ± 5 mmHg, *p* < 0.01; Figure 1B).

### 3.2 SSHTN Increased Bone Loss and Osteoclastogenesis

**FIGURE 2:**
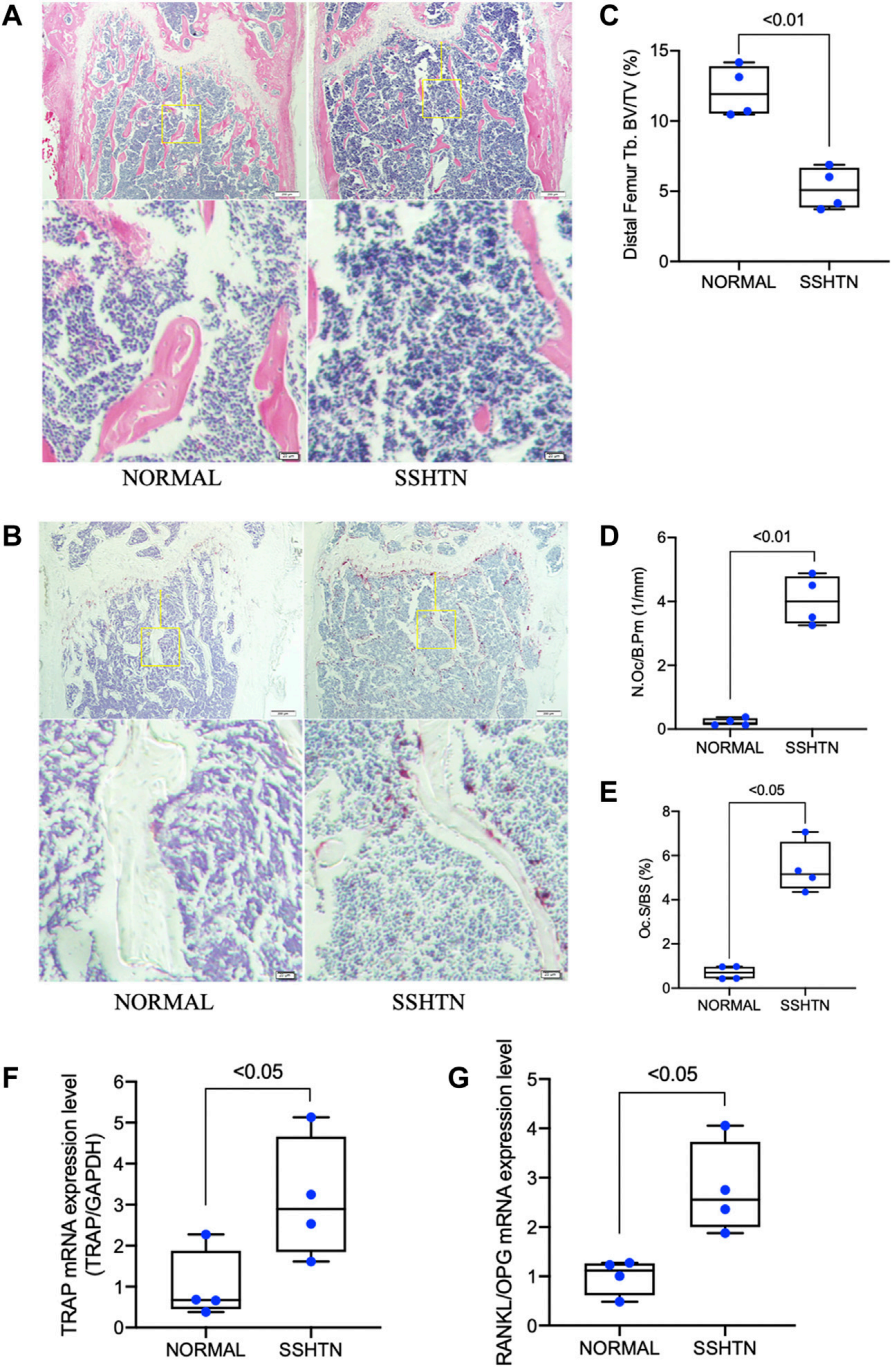
SSHTN was associated with increased bone loss and elevated osteoclastogenesis as determined by histological analysis. Histological sections of decalcified femora from normal control mice or SSHTN mice (*n* = 4) stained with H&E **(A)** and TRAP **(B)**. Scale bar = 200 μm (20 μm in the enlarged picture). **(C)** Quantitative measurement of bone volume fraction (BV/TV). Quantitative measurement of the ratio of **(D)** osteoclast number/bone perimeter (N.Oc/B.Pm) and **(E)** osteoclast surface fraction (Oc.S/BS). **(F)** TRAP mRNA expression level and **(G)** RANKL/OPG mRNA expression level in the tibiae of normal control and SSHTN mice. TRAP, RANKL, and OPG mRNA expression levels were determined by real-time RT-PCR and were normalized relative to GAPDH (*n* = 4).

The effects of SSHTN on osteoclastogenesis and bone loss were analyzed by histomorphometric analyses (Figures 2A,B). H&E staining showed that trabecular bone volume was significantly reduced in SSHTN mice (Figure 2C). In addition, TRAP staining was performed to evaluate several bone parameters to elucidate the effects of SSHTN on osteoclastogenesis *in vivo*. The trabecular distal femur of SSHTN mice showed an increased number of multinucleated TRAP-positive cells (Figure 2D). Consistent with these observations, quantitative histomorphometric analysis showed that Oc.S/BS was elevated in SSHTN mice compared to normal controls (5.43 vs. 0.7%, respectively, *p* < 0.05; Figure 2E). As expected, the mRNA expression level of TRAP, a primary osteoclast marker, was elevated in the tibiae of SSHTN mice compared with normal controls (Figure 2F). RANKL/OPG mRNA expression, important factor for determining osteoclast activation, was also upregulated by hypertension condition (Figure 2G). These results showed that SSHTN resulted in high levels of osteoclastogenesis and increased bone loss.

### 3.3 SSHTN Significantly Increased Bone Resorption Detected on Micro-CT

**FIGURE 3:**
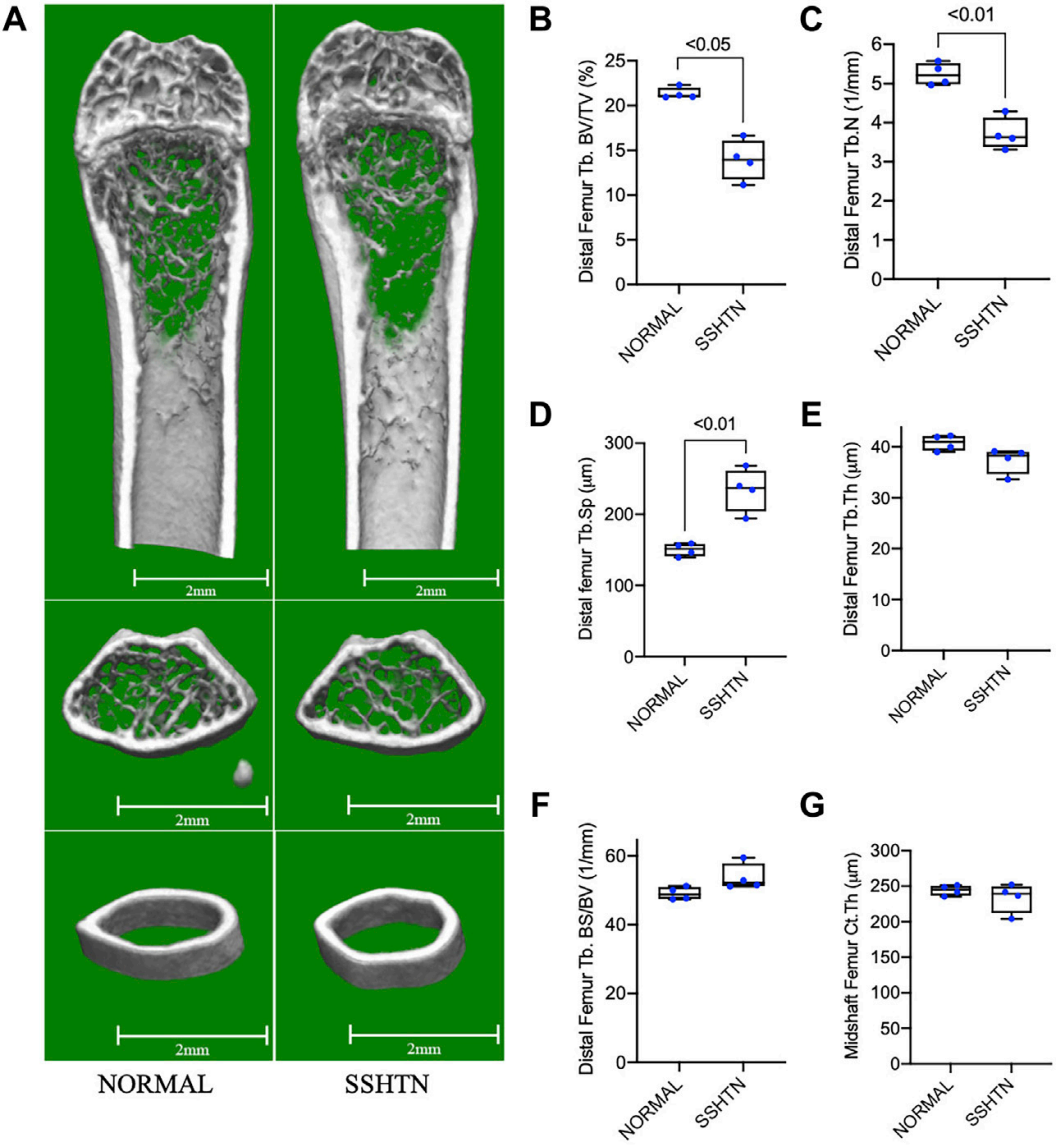
SSHTN mice showed significant elevation of bone resorption determined by micro-CT. **(A)** Micro-CT reconstruction of the trabecular region below the distal femur growth plate and the cortical region of femoral midshaft of normal control or SSHTN mice (*n* = 4). Scale bar = 2 mm. **(B–G)** Quantitative measurement of bone morphometric-related parameters; **(B)** trabecular bone volume fraction (BV/TV), **(C)** trabecular number (Tb.N), **(D)** trabecular separation (Tb.Sp), **(E)** trabecular thickness (Tb.Th), **(F)** trabecular bone surface (BS/BV), and **(G)** cortical thickness (Ct.Th) of normal control and SSHTN mice (*n* = 4).

To examine the effects of SSHTN on bone resorption, we examined the morphometric parameters of femoral bone from normal control and SSHTN mice by micro-CT analysis (Figure 3A). Compared to that of the normal control mice, SSHTN mice showed reductions of 29 and 35% in trabecular number and BV/TV, respectively, while Tb.Sp showed a significant increase by 56% (Figures 3B–D). On the other hand, SSHTN did not have any impact on trabecular thickness, BS/BV, or cortical thickness (Figures 3E–G). These observations confirmed that SSHTN significantly elevated bone resorption, although trabecular and cortical thickness were not altered in these animals.

### 3.4 SSHTN Increased TNF-α and Activated Bone RAS *In Vivo*

**FIGURE 4:**
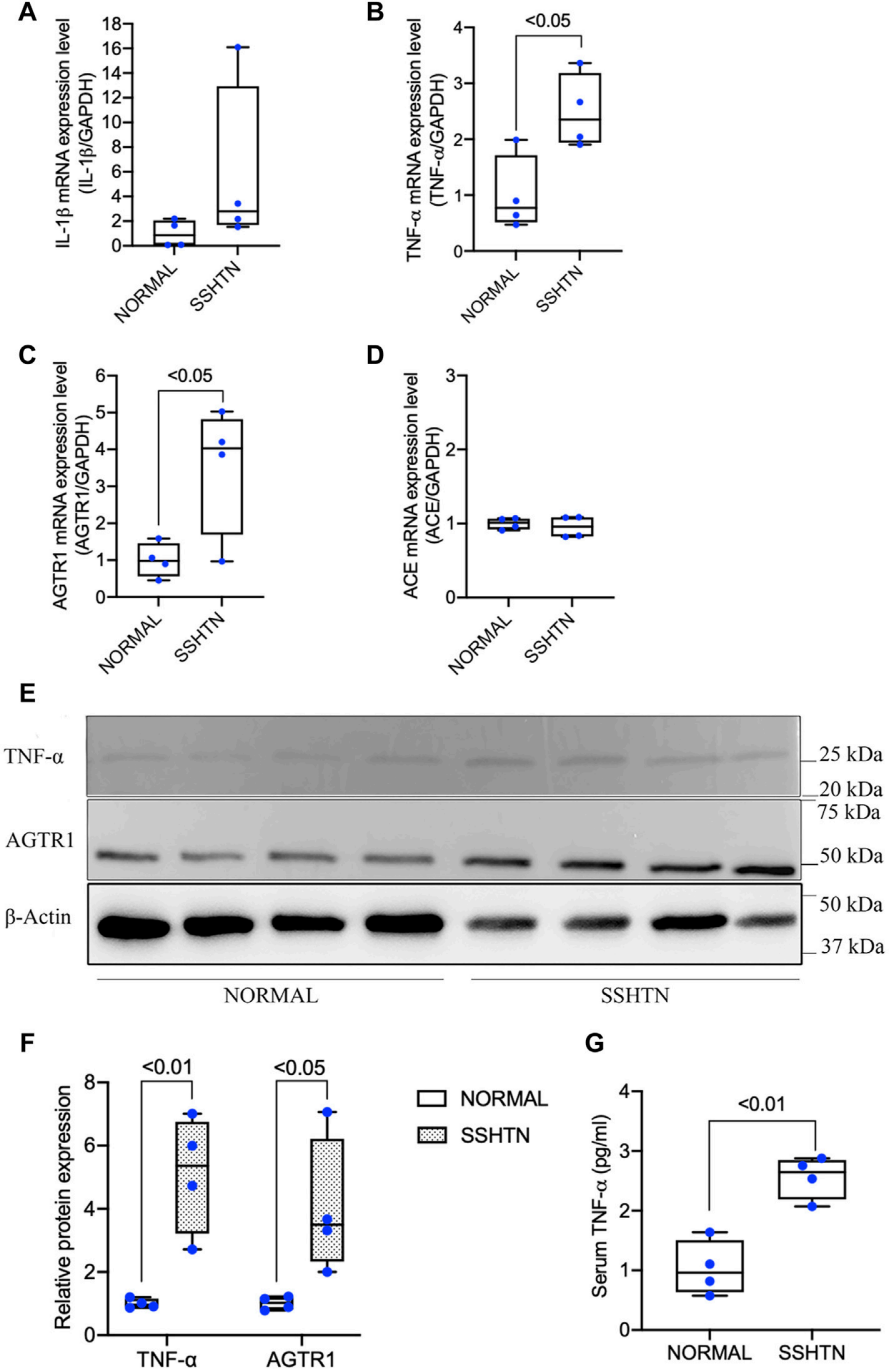
SSHTN mice showed increased TNF-α and activation of bone RAS *in vivo*. **(A)** IL-1β mRNA expression levels in normal control and SSHTN mice. **(B)** TNF-α mRNA expression levels in normal control and SSHTN mice. **(C)** AGTR1 mRNA expression levels in normal control and SSHTN mice. **(D)** ACE mRNA expression levels in normal control and SSHTN mice. IL-1β, TNF-α, AGTR1, and ACE mRNA levels were measured by real-time RT-PCR. Total RNA was isolated from the tibiae of normal control and SSHTN mice. Expression levels of IL-1β, TNF-α, AGTR1, and ACE mRNA were normalized relative to GAPDH (*n* = 4). **(E)** Western blot analysis bands showing the expressions of TNF-α and AGTR1 protein in the distal femur of normal control and SSHTN mice. **(F)** Relative protein expression of TNF-α and AGTR1 were measured using ImageJ software (*n* = 4). β-Actin was used as a loading control. **(G)** Serum levels of TNF-α was determined using ELISA MAX Standard Set Mouse TNF-α. Serum samples were collected from normal control and SSHTN mice (*n* = 4).

As hypertension is considered to be a low-grade inflammatory condition related to the elevation of proinflammatory cytokines, we further examined the effects of SSHTN on proinflammatory cytokine mRNA expression in the tibia in comparison to normal controls. SSHTN stimulated the expression of TNF-α but had no impact on IL-1β mRNA expression (Figures 4A,B). Furthermore, to determine whether the increases in osteoclastogenesis and bone resorption in SSHTN mice could be explained by excessive activation of RAS within the bone, we also investigated AGTR1 and ACE mRNA expression. The results showed that AGTR1 mRNA expression was increased in the tibiae of SSHTN mice, while ACE mRNA expression was similar between normal control and SSHTN mice (Figures 4C,D). Since TNF-α and AGTR1 mRNA expression was increased in SSHTN mice, we also determined the protein expression of TNF-α and AGTR1. As expected, SSHTN upregulated TNF-α and AGTR1 protein expression (Figures 4E,F). In addition, TNF-α serum level was increased in SSHTN mice compare to normal control mice (Figure 4G).

### 3.5 TNF-α Priming Did Not Affect AGTR1 mRNA Expression in Osteoclast Precursors, but Increased AGTR1 mRNA Expression in Osteoblasts Through p38 Activation

**FIGURE 5:**
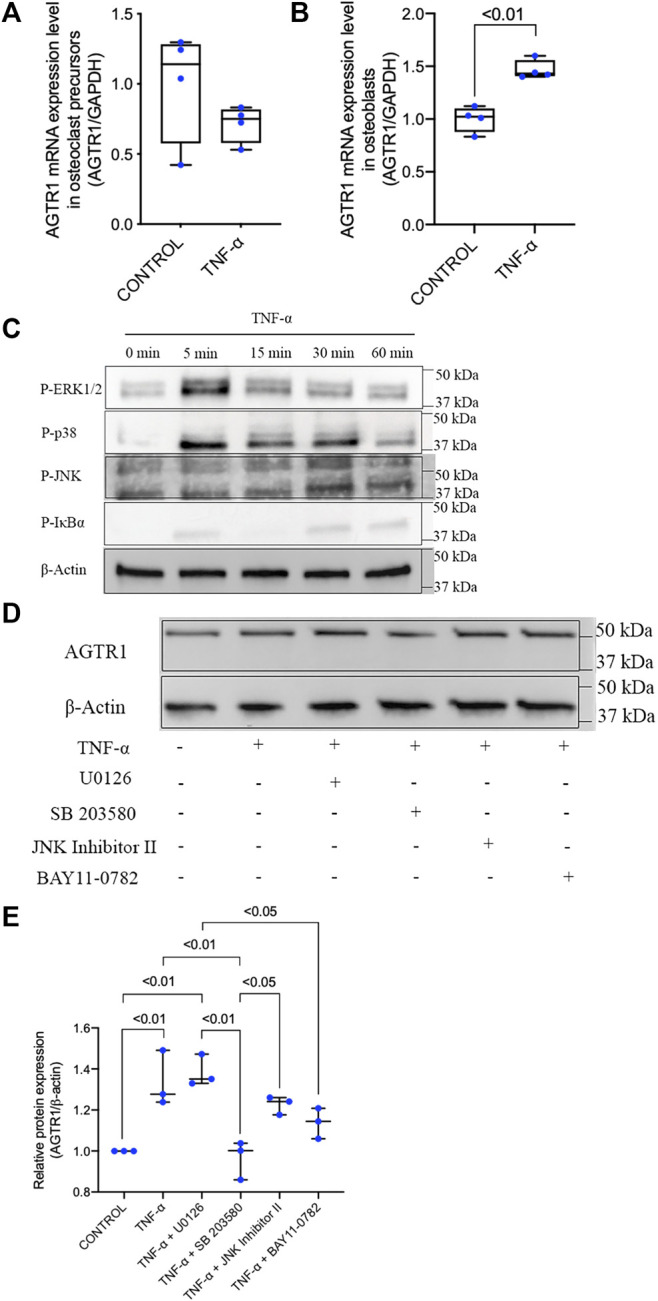
TNF-α had no effect on AGTR1 mRNA expression in osteoclast precursors but increased AGTR1 mRNA expression in osteoblasts through p38 activation. **(A)** AGTR1 mRNA expression level in TNF-α pretreated osteoclast precursors and **(B)** osteoblasts for 24 h. AGTR1 mRNA expression level was determined by real-time RT-PCR. The levels of AGTR1 mRNA expression were normalized relative to GAPDH (*n* = 4). **(C)** Osteoblasts were incubated with TNF-α for 0, 5, 15, 30, or 60 min. Cells were lysed and analyzed by Western blotting using antibodies to phospho-ERK1/2, phospho-p38, phospho-JNK, and phospho-IκBα. β-Actin was used as a loading control. **(D)** Inhibition of p38 signaling prevented induction of AGTR1 protein expression by TNF-α *in vitro*. Osteoblasts were preincubated with or without MAPK and NF-κB inhibitors and then treated with TNF-α for 24 h. Cells were lysed and analyzed by Western blotting using an antibody to AGTR1. **(E)** Relative protein expression of AGTR1 were measured using ImageJ software (*n* = 3). β-Actin was used as a loading control.

The increased levels of both TNF-α and AGTR1 in SSHTN mice suggested that TNF-α may have an effect on AGTR1 expression in bone cells leading to the activation of osteoclasts. Therefore, we examined the effects of TNF-α on osteoclast precursors. Unexpectedly, treatment with TNF-α did not upregulate AGTR1 mRNA expression in osteoclast precursors (Figure 5A). However, stimulation of osteoblasts with TNF-α led to an increase in AGTR1 mRNA expression as determined by real-time RT-PCR (Figure 5B). We further clarified the cellular signaling of TNF-α responsible for upregulation of AGTR1 in osteoblasts. Treatment with TNF-α rapidly increased phosphorylation of ERK1/2, p38, JNK MAPKs, and IκΒα in osteoblasts (Figure 5C). Pretreatment with the p38 MAPK inhibitor, SB 203580, attenuated TNF-α-induced upregulation of AGTR1 protein expression in osteoblasts, whereas the MEK1/2 inhibitor, U0126, JNK Inhibitor II, and NF-κΒ inhibitor, BAY11-0782, showed no such effect (Figures 5D,E). These results suggested a role of the p38 pathway in TNF-α-induced upregulation of AGTR1 protein expression in osteoblasts.

### 3.6 Angiotensin II Increased RANKL/OPG Ratio in TNF-α-Primed Osteoblasts and Enhanced Osteoclastogenesis in TNF-α-Primed Osteoblast/Osteoclast Precursor Co-Culture

**FIGURE 6:**
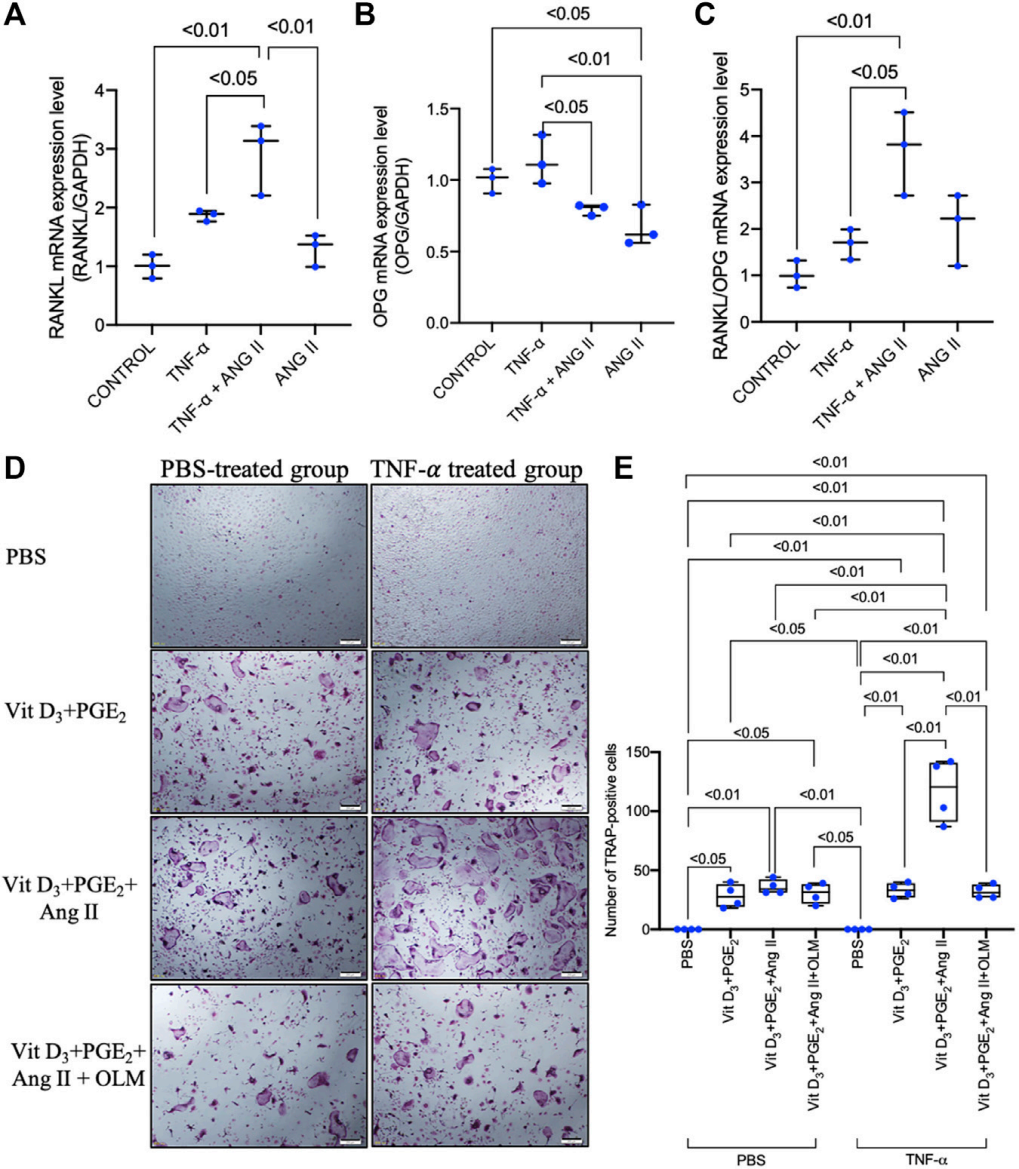
Angiotensin II enhanced the TNF-α-induced increase in RANKL/OPG ratio in osteoblasts and enhanced osteoclastogenesis in TNF-α-primed osteoblast and osteoclast precursor co-culture. **(A)** RANKL mRNA expression level in osteoblasts. **(B)** OPG mRNA expression level in osteoblasts. **(C)** RANKL/OPG mRNA expression levels in osteoblasts. RANKL and OPG mRNA expression levels were determined by real-time RT-PCR and were normalized relative to GAPDH (*n* = 3). **(D)** Micrographs and **(E)** number of large TRAP-positive cells in co-cultures of TNF-α-primed or non-primed osteoblasts and osteoclast precursors treated with PBS, 1.25(OH)_2_D_3_ + PGE_2_, 1.25(OH)_2_D_3_ + PGE_2_ + angiotensin II, and 1.25(OH)_2_D_3_ + PGE_2_ + angiotensin II + angiotensin II type 1 receptor blocker. Scale bar = 200 μm (*n* = 4).

As TNF-α did not induce upregulation of AGTR1 mRNA expression in osteoclast precursors, we further examined whether the increase in AGTR1 mRNA expression level in TNF-α-primed osteoblasts affected the mRNA expression of the osteoclast-related cytokines, RANKL and OPG, resulting in an increase in osteoclast number. Stimulation of TNF-α-primed osteoblasts with 10^–6^ M angiotensin II led to an increase in RANKL mRNA expression (Figure 6A) without significant effect in OPG mRNA expression, as determined by real-time RT-PCR (Figure 6B), resulting in an increase in the RANKL/OPG ratio (Figure 6C). Further analyses using an osteoblast/osteoclast precursor co-culture system showed that angiotensin II enhanced osteoclastogenesis in TNF-α-primed osteoblasts compared to unprimed osteoblasts, whereas cotreatment with an angiotensin II type 1 receptor blocker, olmesartan, completely abolished these effect (Figures 6D,E). Taken together, these results showed that angiotensin II stimulation and increased AGTR1 expression by TNF-α priming increased the RANKL/OPG ratio in osteoblasts leading to osteoclast activation.

### 3.7 SSHTN Exacerbated LPS-Induced Osteoclastogenesis *In Vivo*

**FIGURE 7:**
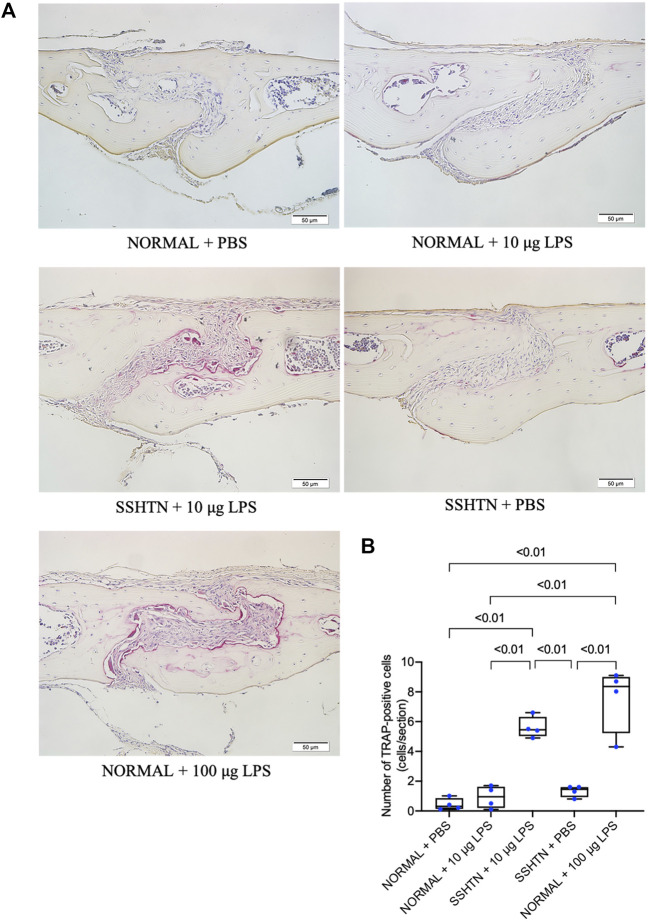
SSHTN mice showed elevated osteoclastogenesis induced by LPS *in vivo*. **(A)** Histological sections of calvariae were obtained from normal control or SSHTN mice after 5 days of daily supracalvarial injection with PBS or LPS. TRAP staining was performed to identify osteoclasts. **(B)** Numbers of multinucleated TRAP-positive cells in the sagittal suture mesenchyme of calvariae were quantified. Scale bar = 50 μm (*n* = 4).

To further examine whether SSHTN enhances LPS-induced osteoclastogenesis, mouse calvariae were subcutaneously injected with PBS or LPS daily for 5 days. Histological sections of calvariae from both normal control and SSHTN mice were stained for TRAP to identify osteoclasts (Figure 7A). The results showed that injection of 100 μg/day LPS in normal control mice resulted in numerous multinucleated TRAP-positive osteoclasts. There were no significant changes in osteoclast number when 10 μg/day LPS was injected into the calvariae of normal control mice and PBS was injected into the calvariae of SSHTN mice. In contrast, 10 μg/day LPS injection in SSHTN mice led to a marked increase in number of multinucleated TRAP-positive osteoclasts (Figure 7B). These observations indicated that the combination of LPS injection and SSHTN led to a further increase in osteoclastogenesis.

### 3.8 SSHTN Exacerbated LPS-Induced Bone Resorption *In Vivo*

**FIGURE 8:**
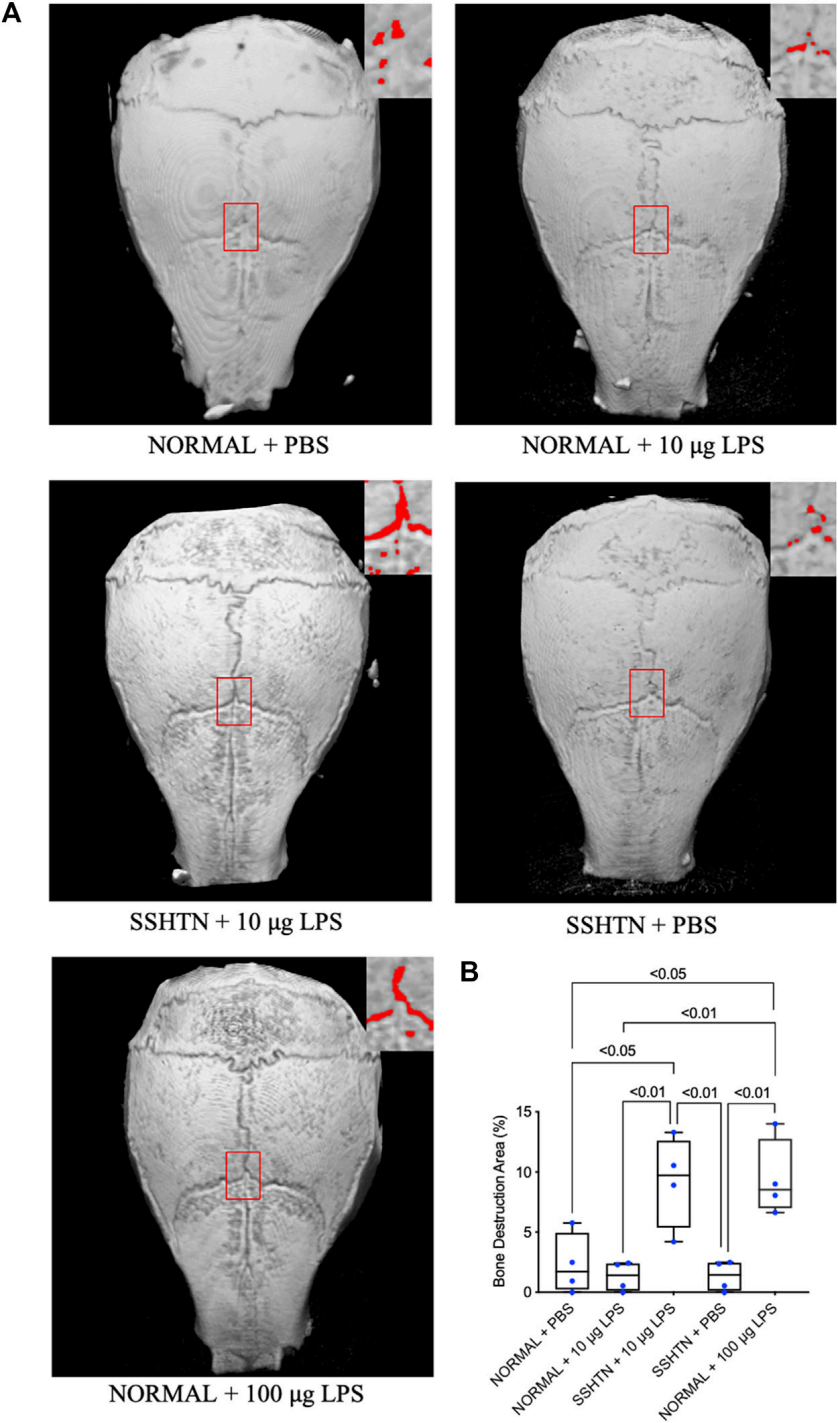
SSHTN mice showed increased bone resorption area induced by LPS *in vivo*. **(A)** Micro-CT reconstructed images of calvariae from normal control or SSHTN mice after daily supracalvarial injection of PBS or LPS for 5 days. Bone resorption areas are shown in red. **(B)** The ratio of bone resorption area to total area was quantified (*n* = 4).

To determine bone resorption area, the mouse calvariae were examined by micro-CT. Figure 8A shows micro-CT images of the calvariae from normal control and SSHTN mice. As shown in Figure 8B, 100 μg/day LPS injection in normal control mice resulted in a significant increase in bone resorption area by 7.1%, while 10 μg/day LPS injection in normal control mice and PBS injection in SSHTN mice did not have any impact on bone resorption area. However, SSHTN mice injected with 10 μg/day LPS showed a marked increase in bone resorption area by 6.9%, suggesting that LPS and SSHTN had a synergistic effect on bone resorption.

## 4 Discussion

It has been difficult to assess the impact of high blood pressure on bone health and bone quality, leading to conflicting reports on hypertension-related bone loss (Tsuda et al., 2001; Javed et al., 2012). In this study, we used post-l-NAME high-salt challenge to generate a mouse model of hypertension to investigate its effects on bone. Our results showed that high blood pressure upregulates osteoclastogenesis, bone resorption, and exacerbates LPS-induced osteoclastogenesis and bone resorption. The distal femora of SSHTN mice showed increased osteoclast number and osteoclast surface fraction, but decreased trabecular bone fraction, which is primarily associated with reduced trabecular number and increased trabecular separation. Consistent with our results, a previous study showed that high blood pressure in spontaneously hypertensive rats (SHR) was associated with significantly reduced bone mineral density and increased risk of fracture with upregulation of markers of bone resorption (Tiyasatkulkovit et al., 2019). Moreover, Dahl salt-sensitive rats also showed osteopenia after chronic salt loading with sodium retention and calcium loss (Titze et al., 2004). Furthermore, recent epidemiological studies demonstrated an association between osteoporosis and high blood pressure. Individuals with higher blood pressure were shown to have increased osteoporotic fracture risk (Li et al., 2017). In addition, a meta-analysis showed that hypertensive women and men have increased bone mineral loss in the lumbar spine, femoral neck, Ward’s triangle, femoral intertrochanteric region, calcaneus, and distal forearm (Ye et al., 2017). Moreover, elevated blood pressure was shown to be associated with decreased bone mineral density (Tiyasatkulkovit et al., 2019; Uchikawa et al., 2019).

To elucidate the mechanisms by which hypertension induces bone resorption, we examined the mRNA expression levels of proinflammatory cytokines and factors and found that SSHTN mice had elevated TNF-α mRNA, protein expression, and serum level in comparison to normal controls. Hypertension is known to be associated with inflammation. Increased serum levels of proinflammatory plasma cytokines, such as CRP, IL-6, IL-1β, and TNF-α, were observed in hypertensive rodent models (Lu et al., 2016; Liu et al., 2019; Dai et al., 2020). In addition, human subjects with high blood pressure were reported to show elevation of circulating proinflammatory cytokine and CRP levels (Jiménez et al., 2016; Pouvreau et al., 2018; Chen et al., 2019; Jayedi et al., 2019). High-salt diet was reported to induce elevation of TNF-α mRNA expression in the paraventricular nucleus and aorta in Dahl salt-sensitive rats, but not in normal controls (Yu et al., 2012; Jiang et al., 2018). Moreover, elevation of proinflammatory cytokines during chronic inflammation has been reported to have a significant effect on bone metabolism, leading to increased risk of bone loss (Baum and Gravallese, 2014; Amarasekara et al., 2015). TNF-α is an effective inducer of osteoclast activity, and has been shown to play important roles in bone metabolism and pathological bone diseases (Kishikawa et al., 2019; Ohori et al., 2020). Therefore, the findings outlined above suggest that TNF-α may have important roles in the processes of bone loss and bone resorption in this SSHTN mouse model.

The systemic RAS is an endocrine system with important roles in regulating blood pressure and electrolyte homeostasis (Ahn et al., 2017; Han et al., 2017). Various organs and tissues, including bone, have also been shown to have local tissue-specific RAS. Moreover, bone RAS overactivation can induce metabolic bone disorders and cause deterioration of bone microcirculation (Shimizu et al., 2008; Asaba et al., 2009; Zhou et al., 2017). ACE and AGTR1 were shown to be highly expressed in the femoral head in an ovariectomized rat model of postmenopausal osteoporosis (Abuohashish et al., 2017b). In addition, the use of AGTR1 antagonists was shown to have therapeutic and protective effects on bone (Abuohashish et al., 2017c; Donmez et al., 2017; Birocale et al., 2019; Dionísio et al., 2020), and ACE inhibitors were shown to have beneficial effects on bone in both experimental and clinical studies (Abuohashish et al., 2017a; Rianon et al., 2017; Chen et al., 2018). Therefore, we also examined whether SSHTN promoted the activation of bone RAS. SSHTN mice showed elevated mRNA and protein expression of the bone RAS component, AGTR1. Indeed, increased AGTR1 mRNA and protein expression were detected in the joint tissues in a transgenic mouse model overexpressing human TNF-α (Akagi et al., 2020). These observations suggest that excessive TNF-α in SSHTN could promote local bone RAS activation, which may have important synergistic effects in SSHTN-induced bone loss.

To further elucidate whether upregulation of TNF-α mRNA expression in SSHTN activates local bone RAS activation resulting in bone loss, we examined the effect of TNF-α on AGTR1 mRNA expression in bone cells. Stimulation with TNF-α did not promote AGTR1 mRNA expression in murine primary osteoclast precursors in culture, while TNF-α enhanced AGTR1 mRNA expression in murine primary osteoblast cultures. TNF-α has been reported to induce AGTR1 expression in cardiac fibroblasts (Peng et al., 2002; Gurantz et al., 2005) and chondrocytes (Tsukamoto et al., 2014). In addition, the transcription factor, NF-κB, and possibly p38 MAPK, were shown to be required for TNF-α-induced AGTR1 upregulation in cardiac fibroblasts (Cowling et al., 2002). Therefore, we also examined the downstream inhibitory effects of TNF-α on ERK1/2, p38, JNK MAPKs, and NF-κB activation using selective inhibitors (U0126, SB 203580, JNK inhibitor II, and BAY11-7082, respectively). However, only the p38 MAPK inhibitor, SB 203580, attenuated AGTR1 protein expression in TNF-α-treated osteoblasts. Angiotensin II has been reported to induce osteoclastogenesis indirectly *via* stromal cells by increasing RANKL expression via AGTR1 receptors (Shimizu et al., 2008). Therefore, we assumed that angiotensin II enhanced TNF-α-induced osteoblast AGTR1 expression and promoted osteoclastogenesis indirectly through TNF-α-mediated osteoblast osteoclastogenic capability. Therefore, we primed osteoblasts with TNF-α followed by treatment with angiotensin II. Stimulation of TNF-α-primed osteoblasts with angiotensin II led to an increase in RANKL/OPG ratio. Furthermore, we also evaluated the effects of TNF-α priming on osteoclastogenesis in osteoblast/osteoclast precursor co-culture and showed that TNF-α priming upregulated osteoclastogenesis compared to untreated controls. The increase in osteoclastogenesis in the TNF-α-primed group was likely due to the increased RANKL/OPG ratio, stimulation by angiotensin II in TNF-α priming osteoblast was shown to markedly increase the RANKL/OPG ratio. These results suggested that angiotensin II stimulation and increased expression of AGTR1 by TNF-α may induce RANKL expression, thus leading to osteoclastogenesis.

It has been reported that subcutaneous administration of LPS at a dose of 100 μg/day for 5 days can promote osteoclastogenesis and bone resorption in calvariae, while daily injection of LPS at 10 μg/day failed to increase osteoclast number and stimulate bone resorption *in vivo* (Ishida et al., 2015; Shima et al., 2018). Consistent with these findings, administration of LPS at a dose of 10 μg/day did not increase osteoclast number *in vivo*. However, SSHTN mice injected with LPS at a dose of 10 μg/day showed an increase in osteoclast number. These observations suggested that SSHTN exacerbates inflammation-induced osteoclastogenesis *in vivo*. In addition, we also examined whether SSHTN exacerbates LPS-induced bone resorption by micro-CT. Increased bone resorption area was only found in normal control mice injected with 100 μg/day LPS and SSHTN mice injected with 10 μg/day LPS, but not in SSHTN mice injected with PBS. These observations suggested that SSHTN exacerbates inflammation-induced osteoclastogenesis and bone resorption, and therefore SSHTN could be a risk factor for progressive bone resorption in inflammatory bone disease.

## 5 Conclusion

The results of the present study demonstrated the detrimental effects of SSHTN on bone health. These hypertensive mice showed deterioration of the bone microstructure, possibly due to increased expression of the proinflammatory cytokine, TNF-α, together with excessive bone RAS activation. Our results may facilitate the development of novel therapeutic strategies to protect bone health under conditions of long-term high-salt intake and hypertension.

## Data Availability

The original contributions presented in the study are included in the article/supplementary material, further inquiries can be directed to the corresponding author.
